# Prevalence of Cognitive Impairment and Its Associated Factors in a Rural Area of Ernakulam District, Kerala, India

**DOI:** 10.7759/cureus.92583

**Published:** 2025-09-17

**Authors:** Vishaka Kiran Jeeje, Gayathri Vimal, Vasanth Bharathidasan, Venkateshan Nithya Veni, Varsha Sudarsanan, Trishaa V, Vinaya Maaniancherry Remesh, Tuhin Dey, Shreya Sreedharan, Aparna Ajay, Akshaya R, Aswathy Sreedevi, Brilly M Rose

**Affiliations:** 1 Community Medicine, Amrita Institute of Medical Sciences and Research Centre, Kochi, IND

**Keywords:** ageing population, cognitive impairement, older adults, physical activity, sociodemographic factors

## Abstract

Introduction

Cognitive impairment is a growing public health concern, particularly among older adults in rural settings. Various factors, including physical activity, comorbidities, and lifestyle behaviors, influence cognitive function. However, evidence on these associations in rural Indian populations remains limited. The objective of this study was to assess the prevalence of cognitive impairment among older adults in a rural region of the Ernakulam district, Kerala, India, and examine its association with sociodemographic and lifestyle factors.

Methods

A community-based cross-sectional study was conducted over two weeks in Njarakkal Grama Panchayat, Ernakulam, Kerala, India, involving 175 participants aged 60 years and above. After obtaining informed consent, data on sociodemographic variables, BMI, physical activity, marital status, and education were collected using a semi-structured questionnaire. Cognitive function was assessed using the Mini-Mental State Examination (MMSE®), and physical activity using the Global Physical Activity Questionnaire (GPAQ). Data were analyzed using Jamovi software (The Jamovi Project, Sydney, Australia). Associations were tested using chi-square or Fisher’s exact tests, and a multivariable logistic regression model was applied to identify independent predictors of cognitive impairment.

Results

The prevalence of cognitive impairment was 27% (95%CI: 20.42%-33.58%). Never-married individuals had significantly higher odds of cognitive impairment compared to those who were ever married (adjusted OR (AOR) = 23.809, 95%CI: 1.445-392.218, p = 0.027). Individuals with only primary or middle school education had substantially higher odds of impairment (AOR = 30.525, 95%CI: 2.817-330.818, p = 0.005). Cognitive impairment was observed in 29.71%, 32.57%, and 37.71% of individuals with high, medium, and low physical activity levels, respectively; however, the association was not statistically significant.

Conclusion

This study highlights a considerable prevalence of cognitive impairment among older adults in rural Ernakulam and identifies significant associations with marital status and educational attainment. Although physical activity showed a graded trend with cognitive outcomes, the relationship was not statistically significant. These findings underscore the importance of addressing social and educational determinants in strategies aimed at preserving cognitive health among the elderly.

## Introduction

The ageing population is rapidly increasing worldwide, bringing with it a surge in age-related health concerns, including cognitive impairment [1,2]. In India, the proportion of the elderly (defined as individuals aged 60 years and above) was approximately 10.1% in 2021, and this is projected to rise to 19.5% by 2050, representing over 300 million people [3]. In Kerala, the situation is even more pronounced, with older adults comprising over 16.5% of the population, the highest among all Indian states [4].

As people age, executive functions tend to decline, sometimes progressing into cognitive impairment. Cognitive impairment is characterised by difficulties in memory, learning, concentration, and decision-making, which can affect day-to-day functioning. It ranges from mild to severe, with mild impairment allowing individuals to function independently, while severe cases result in significant cognitive deficits that hinder communication and independent living [5]. The ageing global population has led to a rising prevalence of mild cognitive impairment (MCI) and subjective cognitive decline [2]. A study conducted in a rural area of Trivandrum, Kerala, reported the prevalence of MCI to be 18.6% among older adults (≥ 65 years) [6]. The role of physical activity and exercise in cognitive impairment among the elderly has been widely debated in studies from other countries.

Physical activity is any movement of the body produced by the contraction of skeletal muscles that results in a substantial increase in caloric requirements over resting energy expenditure. It includes activities such as gardening, shopping, and household chores, whereas exercise refers to planned, structured, and repetitive physical activity aimed at improving or maintaining physical fitness [7].

The impact of physical activity on cognitive health has been extensively studied, with evidence suggesting it enhances cognitive performance through mechanisms such as the stimulation of brain-derived neurotrophic factors and improvements in learning and memory [8]. For older adults, physical activity serves as a non-pharmacological intervention that may mitigate cognitive decline and slow disease-related impairment by targeting neuroprotective mechanisms and modifiable risk factors [9]. These findings underscore the need for further research into cognitive impairment and its associated factors among older populations.

This study aimed to assess the prevalence of cognitive impairment among older adults in a rural region in Kerala, India, and to examine its association with various factors, including physical activity. Understanding these associations may aid in developing strategies to promote cognitive health and well-being among the elderly. Most existing Indian data on cognitive impairment in the elderly are derived from northern populations, while studies from South India remain scarce despite notable differences in lifestyle [10-12]. Hence, this study seeks to address this gap by assessing the prevalence of cognitive impairment and its association with physical activity and other sociodemographic factors among older adults in a rural area of Ernakulam district, Kerala, India.

## Materials and methods

This was a community-based cross-sectional study conducted in Njarakkal Grama Panchayat in the Ernakulam district of Kerala, India. The study was conducted over two weeks, June 1-14, 2024. The study was approved by the Ethics Committee of Amrita School of Medicine, Kochi, Kerala, India (approval number: ECASM-AIMS-2024-151). Informed consent was obtained both verbally and in writing. The study was explained in detail to each participant in Malayalam using a translated participant information sheet and consent form. After confirming that the participant understood the study procedures, written consent was obtained through the participant's signature.

Study population

The study population comprised elderly individuals aged 60 years and above residing in the selected area of Njarakkal Grama Panchayat. Individuals were included if they were aged 60 years or older, were permanent residents of the panchayat, and provided informed consent to participate. Individuals with known stroke-related cognitive disabilities, significant hearing loss, or previously diagnosed psychiatric or intellectual disabilities were excluded from the study.

Sample size

Based on a study by Kumari et al. [13], which reported a 36% prevalence of cognitive impairment, the sample size was calculated using the formula: $n = \frac{\left(Z_{1-\alpha/2}\right)^{2} \, p \, (1 - p)}{d^{2}}$= 1.96 (for 95%CI), p = 0.36, and d = 0.08 (absolute precision). After adjusting for a 10% non-response rate, the final sample size was calculated as 155. However, data were ultimately collected from 175 individuals.

Sampling technique

A consecutive sampling method was employed to recruit participants. All eligible individuals aged 60 years and above residing in Njarakkal Grama Panchayat were approached through house-to-house visits during the two-week study period. Those who met the inclusion criteria and provided informed consent were enrolled until the required sample size was reached. This approach ensured comprehensive coverage of the elderly population in the selected area and minimized selection bias within the time constraints of the study.

Study tools

Sociodemographic data were collected using an interview schedule that incorporated multiple standardized instruments. Sociodemographic and lifestyle information, including variables such as name, age, sex, body mass index (BMI), marital status, education level, occupation, alcohol and tobacco use, and comorbidities, was collected using a semi-structured questionnaire. 

Cognitive function was assessed using the Mini-Mental State Examination (MMSE) questionnaire [14]. The MMSE is a copyrighted instrument and may not be used or reproduced in whole or in part, in any form or language, or by any means without written permission of PAR (www.parinc.com). The MMSE evaluates five cognitive domains: orientation, registration, attention and calculation, recall, and language, with a total score of 30. Participants scoring ≤ 23 were classified as cognitively impaired, while those scoring > 23 were considered cognitively normal.

Physical activity levels were measured using the Global Physical Activity Questionnaire (GPAQ) [15], which quantifies activity in terms of metabolic equivalence (MET). One MET corresponds to the energy expenditure of sitting quietly (1 kcal/kg/hour). Physical activity was categorized based on total MET minutes per week, with < 600 MET-minutes/week classified as low activity, 600-3000 MET-minutes/week as moderate activity, and > 3000 MET-minutes/week as high activity. To calculate total MET minutes, the time spent in moderate and vigorous activities was converted to minutes, multiplied by their respective MET values (4 for moderate, 8 for vigorous), and summed across all domains. The GPAQ is a publicly available tool developed by the World Health Organization, which does not require prior permission for non-commercial research use.

All interviews were conducted face-to-face by trained medical student investigators, who are part of this study. No self-administered questionnaires were used. The tools were administered orally to accommodate participants with varying levels of literacy and to ensure full comprehension and consistency in data collection.

Data collection

Data were collected by trained medical student investigators under the supervision of the faculty guide. A paper-based data collection method was used, and forms were manually filled during interviews. The collected data were later entered into Microsoft Excel (Microsoft Corporation, Redmond, Washington, United States) by the student researchers for further analysis. Double data entry and verification were performed to ensure accuracy.

Statistical analysis

Data were entered into Microsoft Excel and analyzed using Jamovi software version 2.5.6 (The Jamovi Project, Sydney, Australia; https://www.jamovi.org/). Categorical variables were summarized using frequencies and percentages, while continuous variables were presented as mean ± standard deviation (SD). Cognitive status (normal vs. impaired) was reported in terms of frequency and percentage. Associations between cognitive impairment and potential influencing factors, including physical activity levels, were assessed using the chi-square test or Fisher's exact test, as appropriate. A multivariable logistic regression analysis was conducted using the backward logistic regression method to determine the most significant sociodemographic predictors of cognitive impairment. 

## Results

The mean age of the study population was 68.1 ± 6.21 years. Based on sex distribution, more than half were female, constituting 56.6%. The sociodemographic characteristics of the participants are mentioned in the table below. (Table 1)

**Table 1:** Sociodemographic and lifestyle factors of the study population (N=175) BMI: body mass index; APL: above poverty line; BPL: below poverty line NOTE: “joint family” excludes “three-generation family" in this table

Variables	Category	Frequency	Percentage
Age (in years)	≤68	97	55.4
	>68	78	44.6
Sex	Female	76	43.4
	Male	99	56.6
Type of family	Nuclear family	74	42.3
	Joint family	7	4.0
	3-generation family	94	53.7
Marital status	Never married	50	28.6
	Married at least once	125	71.4
Education	Primary/Middle	69	39.4
	High school/higher secondary	85	48.6
	University	21	12
Occupation	Skilled	19	10.9
	Unskilled	26	14.9
	Retired/professional	65	37.1
	Homemaker/Unemployed	65	37.1
Socioeconomic status	APL	112	64
	BPL	63	36
Substance use	Drinking only	19	10.9
	Smoking only	4	2.3
	Drinking & Smoking	14	8.0
	Tobacco chewing	2	1.1
	No habits	136	77.7
BMI (kg/m^2^)	Underweight (<18.5)	14	8.0
	Normal (18.5 - 22.9)	61	35
	Overweight (>22.9)	100	57

The overall prevalence of cognitive impairment in the study population was 27% (95%CI 0.220, 0.320), with a mean MMSE score of 25.4 ± 4.31. Among the participants, 29.71% (n=52) had high physical activity levels, 32.57% (n=57) had medium activity levels, and 37.71% (n=66) had low physical activity levels.

On performing univariate analysis to examine the association between cognitive impairment and various sociodemographic and lifestyle variables, including physical activity, several significant associations were observed. Male participants (76.3%) were found to have a significantly higher prevalence of cognitive impairment compared to female participants (61.6%). Marital status was also significantly associated with cognitive function, with unmarried individuals showing a higher prevalence of cognitive impairment (52%) compared to their married counterparts (16.8%). Educational level demonstrated a strong association (p < 0.001), with only 4.8% of university-educated individuals experiencing cognitive impairment, whereas the prevalence was markedly higher (47.8%) among those with only primary or middle school education. Socioeconomic status, assessed based on ration card type, was significantly associated with cognitive impairment (p = 0.004), as individuals holding below poverty line (BPL) cards exhibited a higher prevalence (39.7%) than those with above poverty line (APL) cards (19.6%). Body mass index (BMI) also showed a significant association (p = 0.026), with underweight individuals (50%) exhibiting a higher prevalence of cognitive impairment compared to those who were of normal weight (32.8%) or overweight (20%) (Table 2).

**Table 2:** Univariate analysis to test for association between MMSE® scores with various sociodemographic variables and other lifestyle factors # Fisher’s exact test APL: above poverty line; BPL: below poverty line The MMSE is a copyrighted instrument and may not be used or reproduced in whole or in part, in any form or language, or by any means without the written permission of PAR (www.parinc.com).

Sociodemographic factors		Cognitive Impairment		p-value
		No cognitive impairment, n (%)	Cognitive impairment present, n (%)	
Age (in years)	≤68	74 (76.3%)	23 (23.7%)	0.295
	>68	54 (69.2%)	24 (30.8%)	
Sex	Male	38 (38.4%)	61 (61.6%)	0.039
	Female	18 (23.7%)	58 (76.3%)	
Type of family	Nuclear	56 (75.7%)	18 (24.3%)	0.792#
	Joint family	5 (71.4%)	2 (28.6%)	
	3-generation family	67 (71.3%)	27 (28.7%)	
Marital status	Never married	24 (48%)	26 (52%)	<0.001
	Married at least once	104 (83.2%)	21 (16.8%)	
Education	Primary/Middle	36 (52.2%)	33 (47.8%)	<0.001#
	High school/Higher secondary	72 (84.7%)	13 (15.3%)	
	University	20 (95.2%)	1 (4.8%)	
Occupation	Skilled	16 (84.2%)	3 (15.8%)	0.146#
	Unskilled	16 (61.5%)	10 (38.5%)	
	Retired/professional	44 (67.7%)	21 (32.3%)	
	Homemaker/Unemployed	52 (80%)	13 (20%)	
Ration card	APL	90 (80.4%)	22 (19.6%)	0.004
	BPL	38 (60.3%)	25 (39.7%)	
Substance use	Yes	31 (79.5%)	8 (20.5%)	0.311
	No	97 (71.3%)	39 (28.7%)	
BMI (Kg/m^2^)	Normal	41 (67.2%)	20 (32.8%)	0.026
	Underweight	7 (50%)	7 (50%)	
	Overweight	80 (80%)	20 (20%)	
Physical activity levels	High	10 (21.3%)	42 (32.8)	0.228
	Moderate	15 (31.9%)	42 (32.8%)	
	Low	22 (46.8%)	44 (34.4%)	

A multivariable logistic regression analysis was conducted using the backward logistic regression method to determine the most significant sociodemographic predictors of cognitive impairment. Adjusted odds ratios (AOR) with 95%CI were computed to assess the independent effects of various factors (Table 3).

**Table 3:** Factors influencing MMSE® score levels: multivariable logistic regression The MMSE is a copyrighted instrument and may not be used or reproduced in whole or in part, in any form or language, or by any means without the written permission of PAR (www.parinc.com).

Variable	Category	AOR	95% CI		p-value
			Lower	Upper	
Marital status	Never married	23.809	1.445	392.218	0.027
	Married at least once	1			
Education	Primary/Middle	30.525	2.817	330.818	0.005
	High school/higher secondary	5.311	0.489	57.676	0.170
	University	1			

Marital status and education emerged as significant independent predictors of cognitive impairment in the multivariable analysis. Individuals who were never married had significantly higher odds of cognitive impairment compared to those who were ever married (AOR = 23.809, 95%CI: 1.445-392.218, p = 0.027). Similarly, education level was a strong predictor, with individuals who had only primary or middle school education showing markedly higher odds of cognitive impairment (AOR = 30.525, 95%CI: 2.817-330.818, p = 0.005) compared to those with higher secondary or university education. In contrast, socioeconomic status and BMI were not found to be significantly associated with cognitive impairment after adjusting for other variables in the multivariable model.

## Discussion

This study highlights several key sociodemographic and lifestyle factors associated with cognitive impairment among the elderly. A higher prevalence of cognitive impairment was observed among males, unmarried individuals, and those with lower levels of educational attainment. Notably, male participants exhibited a greater degree of cognitive decline compared to females, and individuals who had never married were at higher risk than their counterparts who had married at least once. Educational status emerged as a strong determinant, with those having only primary or middle school education demonstrating a greater likelihood of cognitive impairment than individuals with higher secondary or university-level education.

The overall prevalence of cognitive impairment in the present study was 27% (95% CI: 0.220-0.320), which is substantially higher than figures reported in earlier Indian studies. For instance, a study from rural Trivandrum documented an age-standardised prevalence of 18.6% among the elderly [6]. This elevated prevalence underscores the growing concern of cognitive impairment among rural elderly populations and may reflect gaps in awareness, underdiagnosis, or contextual differences in lifestyle and healthcare access.

A noteworthy finding of our study was the significant association between marital status and cognitive function. Individuals who were ever married demonstrated a lower prevalence of cognitive impairment, highlighting the potential protective role of social support. This aligns with the findings of Lee et al., who suggested that marriage fosters cognitive health through companionship, emotional connection, and participation in mentally stimulating activities [16]. Supporting evidence also links social isolation, emotional neglect, and reduced cognitive engagement-conditions more common among those living alone-with a heightened risk of cognitive impairment [17,18]. Thus, marriage may serve as an important source of psychosocial stimulation, potentially mitigating cognitive decline.

The influence of educational qualification on cognitive health was supported by our findings. Participants with lower educational levels, particularly those with only primary or middle school education, were more prone to cognitive impairment. These results are consistent with studies by Cabero Castro et al. [19] and Zhong et al. [20], which demonstrated that higher educational attainment contributes to greater cognitive reserve and improved performance in domains such as memory, attention, and reasoning. A cohort study in China similarly concluded that cognitive reserve, developed through lifelong education and active social and intellectual engagement, was associated with longer disability-free survival [21]. Furthermore, a seven-year longitudinal study in China observed that older adults with higher cognitive reserve exhibited better baseline cognitive performance and a slower rate of cognitive decline, particularly in rural settings [22]. These findings collectively underscore the importance of lifelong learning and cognitive engagement in preserving mental acuity with aging.

Although physical activity is widely acknowledged for its role in preventing chronic diseases and reducing mortality [23], our study did not find a statistically significant association between physical activity and cognitive function. This discrepancy may be attributed to various factors, including the type, intensity, and consistency of physical activity, as well as genetic predisposition, socioeconomic conditions, behavioral traits, and age-related variations [24-26]. While low-intensity or irregular activity may not substantially influence cognitive outcomes, it can still contribute to improved physical health and overall well-being [27]. Specifically, aerobic exercise has been shown to positively affect cognitive function [28]. Despite the lack of statistical significance in our findings, the observed trends suggest that physical activity remains a promising modifiable factor. Further longitudinal and interventional studies in similar rural populations are warranted to explore this relationship more comprehensively.

This study is not without limitations. Although a consecutive sampling method was used to minimize selection bias, the short duration of data collection and reliance on available participants may still limit the generalisability of the findings. Additionally, the use of self-reported physical activity data through the GPAQ may be prone to recall and reporting bias, particularly among older participants.

## Conclusions

This study identified a 27% prevalence of cognitive impairment among older adults in rural Ernakulam, with significant associations observed for sociodemographic factors such as marital status and educational attainment. These findings underscore the growing burden of cognitive impairment among the elderly in rural Kerala, reflecting broader demographic and social transitions. The protective associations of being married and having higher educational qualifications highlight the critical role of sustained social interaction and cognitive engagement in promoting healthy ageing.

The findings call for a multidimensional approach to cognitive health promotion, one that moves beyond medical interventions to incorporate educational, social, and behavioural strategies. Community-based initiatives that foster social inclusion, encourage literacy and lifelong learning, and promote physical activity could serve as sustainable interventions in resource-constrained rural settings. Furthermore, the study highlights the need for strengthening local health systems to incorporate routine cognitive screening and counselling services. Longitudinal and interventional studies are essential to establish causality and to develop evidence-based policies aimed at preserving cognitive function and improving the quality of life of the ageing population.
